# Hematopoietic lineage-converted T cells carrying tumor-associated antigen-recognizing TCRs effectively kill tumor cells

**DOI:** 10.1136/jitc-2019-000498

**Published:** 2020-07-14

**Authors:** Fangxiao Hu, Dehao Huang, Yuxuan Luo, Peiqing Zhou, Cui Lv, Kaitao Wang, Qitong Weng, Xiaofei Liu, Yuxian Guan, Yang Geng, Juan Du, Jiekai Chen, Jinyong Wang, Hongling Wu

**Affiliations:** 1 School of Life Sciences, University of Science and Technology of China, Hefei, Anhui, China; 2 CAS Key Laboratory of Regenerative Biology, Guangzhou Institutes of Biomedicine and Health, Chinese Academy of Sciences, Guangzhou, Guangdong, China; 3 Guangdong Provincial Key Laboratory of Stem cell and Regenerative Medicine, Guangzhou Institutes of Biomedicine and Health, Chinese Academy of Sciences, Guangzhou, Guangdong, China; 4 University of Chinese Academy of Sciences, Beijing, China; 5 Department of Pediatrics, Guangzhou Women and Children's Medical Center, Guangzhou, Guangdong, China; 6 Joint School of Life Sciences, Guangzhou Medical University, Guangzhou, Guangdong, China; 7 Guangzhou Regenerative Medicine and Health-Guangdong Laboratory (GRMH-GDL), Guangzhou, Guangdong, China; 8 Institute for Stem Cell and Regeneration, Chinese Academy of Sciences, Beijing, China

**Keywords:** T lymphocytes, melanoma, immunotherapy

## Abstract

Tumor-associated antigen (TAA) T-cell receptor (TCR) gene-engineered T cells exhibit great potential in antitumor immunotherapy. Considering the high costs and low availability of patient-derived peripheral blood T cells, substantial efforts have been made to explore alternatives to natural T cells. We previously reported that enforced expression of *Hoxb5* converted B cells into induced T (iT) cells *in vivo*. Here, we successfully regenerated naive OT1 (major histocompatibility complex I restricted ovalbumin antigen) iT cells (OT1-iT) *in vivo* by expressing *Hoxb5* in pro-pre-B cells in the OT1 transgenic mouse. The OT1-iT cells can be activated and expanded *in vitro* in the presence of tumor cells. Particularly, these regenerated OT1-iT cells effectively eradicated tumor cells expressing the TAA (ovalbumin) both *in vitro* and *in vivo*. This study provides insights into the translational applications of blood lineage-transdifferentiated T cells in immunotherapy.

## Introduction

Tumor-associated antigen (TAA) T-cell receptor (TCR) gene-engineered T cell (TAA-TCR-T) therapy has shown great prospect in treating malignant cancers such as melanoma, sarcoma, mesothelioma, and other malignancies.^1 2^ Numerous research groups have been focusing on preparing high-avidity TCRs of TAA, and maintaining the T-cell activity and longevity *in vitro* during stimulation and expansion.^3–5^ In regenerative medicine, it has been a central aim to produce cellular alternatives to natural peripheral blood (PB) T cells. One conventional attempt is to deliver tumor-specific TCR genes into the hematopoietic stem cells (HSCs), which can differentiate into antitumor T cells.^6 7^ However, this approach contains the risk of a patient sustainably producing TAA-TCR-T cells throughout their lifespan, as well as the potential contamination of TCR expression in other blood lineage cells. Recently, scientists have turned their emphasis on induced pluripotent stem cells (iPSCs), as TAA-TCRs can be introduced into iPSCs to form TAA-TCRs-iPSC clones without compromising the key traits of these stem cells.^8–10^ Nonetheless, a fast method of regenerating TAA-TCR *in vivo* remains elusive.

Blood lineages can be regenerated by direct lineage transdifferentiation approaches.^11–14^ Recently, we reported that B cells can be converted into functional T cells by Hoxb5 protein, a transcription factor that is not expressed in B cells nor in T cells.^15^ Here, we translationally extended our study and regenerated TAA-TCR induced T (iT) cells by manipulating the OT1 pro-pre-B cells sorted from the OT1 transgenic mouse using a retrovirus delivery system expressing the *Hoxb5 in vivo*. Major histocompatibility complex I (MHC-I) restricted CD8^+^ OT1-iT cells were successfully regenerated in the peripheral immune organs of the recombination activating gene 1 mutation (*Rag1^-/-^)* recipients, a mouse strain lacking natural T and B cells. *In vitro* and *in vivo* functional assays provide robust evidence that the regenerated TAA-TCR-iT cells have the capacity of specifically killing tumor cells expressing the TAA. Regarding the short-time window, transiency, perfect development of iT regeneration process *in vivo* by B-to-T lineage transdifferentiation,^15^ we document a *de novo* alternative approach to regenerate TAA-TCR iT cells by blood lineage transdifferentiation *in vivo*.

## Results

### Ectopic expression of the *Hoxb5* reprogrammed OT1 B cells into OT1-iT cells

**Figure 1:**
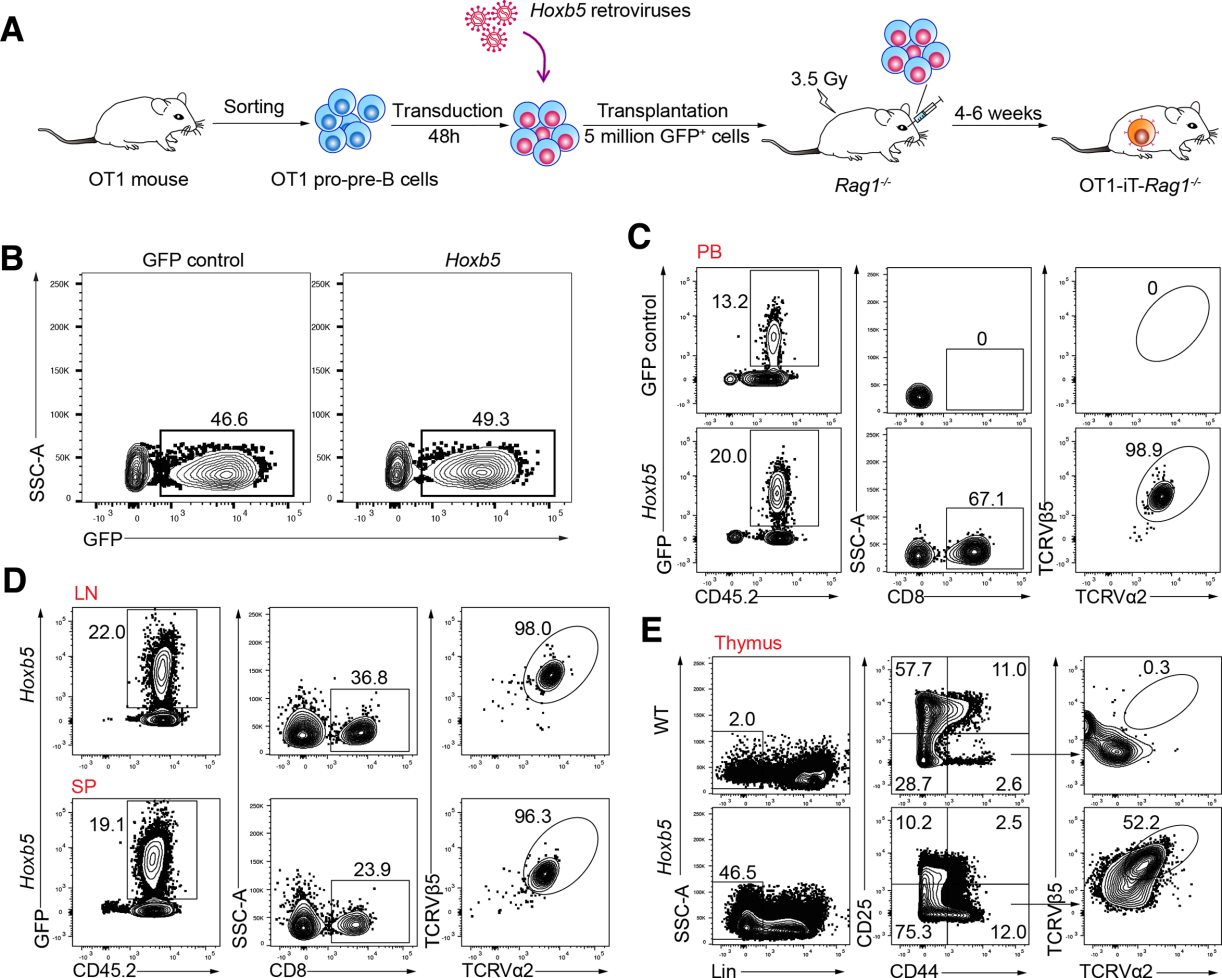
Immunophenotypic characterization of the OT1-iT cells. (A) Schematic strategy of generating OT1-iT by ectopic expression of *Hoxb5* retroviruses in OT1 pro-pre-B cells. OT1 pro-pre-B cells were sorted from bone marrow-nucleated cells from OT1 transgenic mouse (C57BL/6 mouse strain), transduced with the *Hoxb5* retroviruses, and subsequently transplanted into irradiated *Rag1^-/-^* mice (3.5 Gy, 5 million GFP^+^ cells per mouse, OT1-iT-*Rag1^-/-^*). (B) The transduction rates of the OT1 pro-pre-B cells infected with either the *Hoxb5* or GFP control retroviruses. *Hoxb5* retroviruses or GFP control retroviruses were transduced into the OT1 pro-pre-B cells (GFP control or *Hoxb5*) by two rounds of spin transfection. The GFP-positive population indicated the infected OT1 pro-pre-B cells. (C) Flow cytometric analysis of the mature OT1 iT cells in the PB of the OT1-iT-*Rag1^-/-^* mouse 4 weeks’ post-transplantation. OT1-positive iT cells were defined as CD45.2^+^GFP^+^CD8^+^TCRVα2^+^TCRVβ5^+^. Representative plots from recipients of GFP control OT1 pro-pre-B (GFP control) and *Hoxb5* OT1 pro-pre-B (*Hoxb5*) are shown. (D) Flow cytometric analysis of the mature OT1 iT cells in the LN and SP of the OT1-iT-*Rag1^-/-^* mice (*Hoxb5*) 4 weeks’ post-transplantation. (E) Intracellular staining of the expression of the OT1 in the donor-derived thymocytes. DN1 cells with the expression of the OT1 were defined as CD45.2^+^GFP^+^Lin (CD4, CD8, B220, Gr1, Mac1, Ter119)^-^CD44^+^CD25^-^TCRVα2^+^TCRVβ5^+^. Flow plots of one representative recipient (*Hoxb5*) and WT control mouse are shown. SSC-A, side scatter area; DN1, stage 1 double-negative thymocytes; iT, induced T cells; LN, lymph nodes; PB, peripheral blood; SP, spleen; TCR, T-cell receptor; WT, wild type.

To produce OT1-iT cells converted from the OT1 pro-pre-B cells, we sorted OT1 pro-pre-B cells (CD3^-^Mac1^-^Ter119^-^B220^+^CD19^+^CD93^+^IgM^-^) from the bone marrow nucleated cells of OT1 C57BL/6 transgenic mice and transduced them with *Hoxb5* retroviruses or green fluorescent protein (GFP) control following a previous protocol.^16^ Next, the transduced cells were retro-orbitally transplanted into sublethally irradiated *Rag1^-/-^* mice (C57BL/6, 3.5 Gy, 5 million cells/mouse) to generate the OT1-iT cells (online supplementary figure S1a; figure 1A, B). Four to six weeks post-transplantation, the OT1-iT cells appeared in the PB, lymph node (LN), and spleen (SP) of the recipient OT1-iT-*Rag1^-/-^* mice (figure 1C, D). Additionally, the OT1-TCR proteins were expressed on the surface of the stage 1 double-negative thymocytes (DN1 cells) in the thymus of the OT1-iT-*Rag1^-/-^* mice (figure 1E). As expected, there were no iT generated in the PB of the Rag1^-/-^ recipients transplanted with GFP control transduced pro-pre-B cells (figure 1C). To validate that the OT1-iT cells were derived from the OT1 pro-pre-B cells rather than natural OT1 T-cell contaminants, we performed DNA sequencing of B cell receptor (BCR) heavy chain (IgH) rearrangements using the genome from the single OT1-iT cells which were sorted from the SP of the OT1-iT-*Rag^-/-^* mouse using a previously reported protocol.^15^ As expected, the single OT1-iT cells contained B-cell antigen receptor immunoglobulin heavy-chain V(D)J rearrangements (online supplementary figure S1b), which signaled their B cell origin. Furthermore, donor-derived Lin^-^Sca1^+^c-kit^+^ (LSK) and common lymphoid progenitor (CLP) cells were absent in the bone marrow of the recipients’ 6 weeks’ post-transplantation (online supplementary figure S1c), which further excludes the possibility of donor long-term HSC contamination. Collectively, these results indicate that OT1 pro-pre-B cells can be converted into OT1-iT cells in the presence of *Hoxb5*.

10.1136/jitc-2019-000498.supp1 Supplementary data

### OT1-iT cells specifically kill B16F10-OVA tumor cells *in vitro*

**Figure 2:**
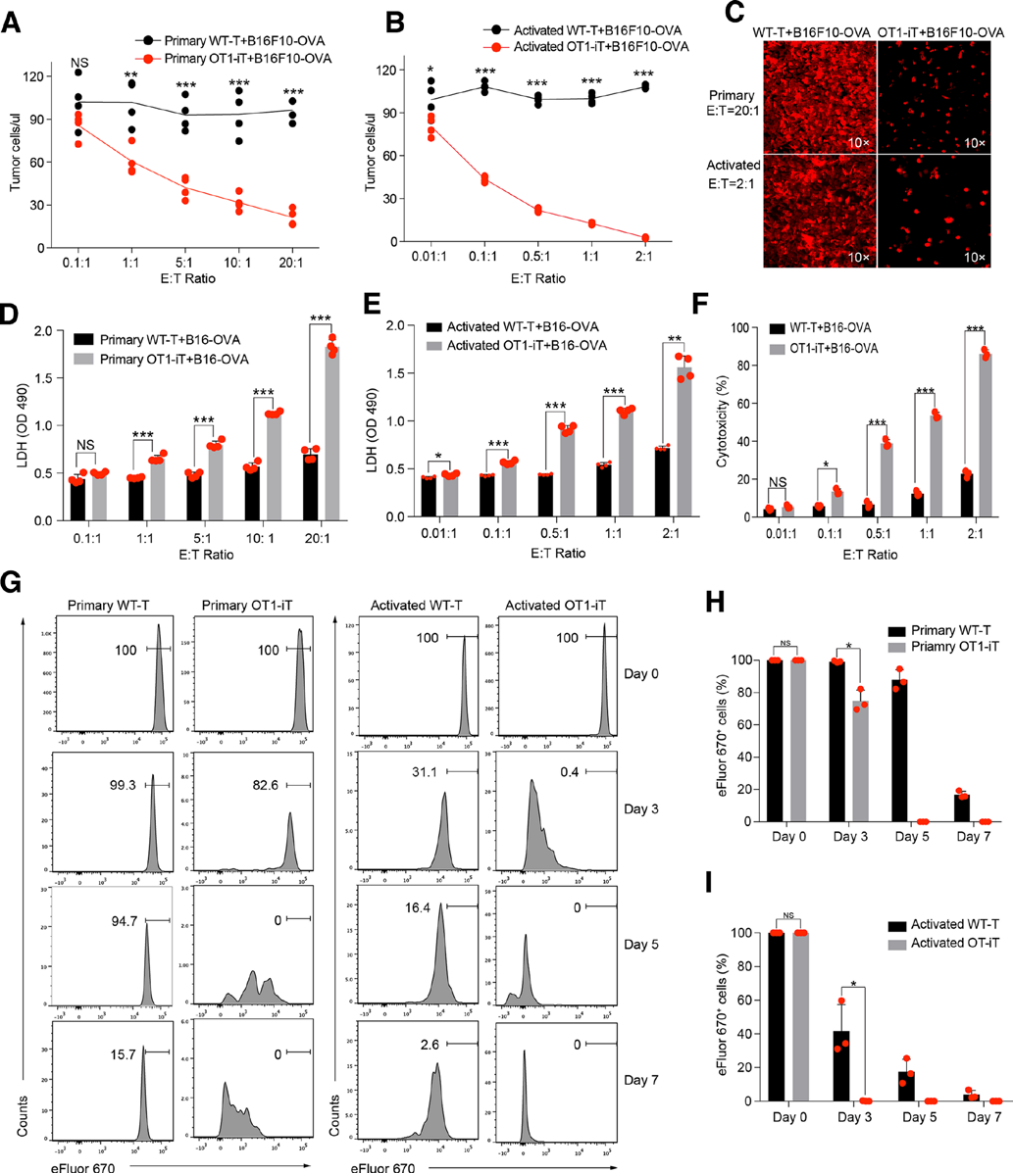
OT1-iT cells exhibit antitumor activity *in vitro*. Primary OT1-iT cells were enriched from the spleens of the OT1-iT-*Rag1^-/-^* mice 6 weeks’ post-transplantation. Activated OT1-iT cells were obtained by stimulating primary OT1-iT cells with anti-CD3/CD28 beads for 4 days. (A) Cell number of the B16F10-OVA tumor cells after 36 hours of coculture with primary OT1-iT cells. A total of 1×10^4^ B16F10-OVA tumor cells as target (T) cells were cocultured with primary OT1-iT (1×10^3^, 1×10^4^, 5×10^4^, 1×10^5^, and 2×10^5^) or control WT-T cells as effector (E) cells for 36 hours at different E:T ratios (0.1:1, 1:1, 5:1, 10:1, 20:1) (n=4). (B) The number of B16F10-OVA tumor cells after 36 hours co-culture with activated OT1-iT cells. A total of 1×10^4^ B16F10-OVA tumor cells as target (T) cells were co-cultured with activated OT1-iT (1×10^2^, 1×10^3^, 5×10^3^, 1×10^4^, and 2×10^4^) as effector (E) cells for 36 hours at different E:T ratios (0.01:1, 0.1:1, 0.5:1, 1:1, 2:1) (n=4). (C) Representative microphotograph of primary and activated OT1-iT cells cocultured with B16F10-OVA tumor cells at the indicated E:T ratio for 36 hours. (D) LDH release of the B16F10-OVA tumor cells after 36 hours’ coculture with primary OT1-iT cells or control WT-T cells (n=4). (E) LDH release of the B16F10-OVA tumor cells after 24 hours’ coculture with activated OT1-iT cells or control WT-T cells (n=4). (F) Cytotoxicity of the OT1-iT cells or control WT-T cells cocultured with B16F10-OVA tumor cells for 24 hours (n=3). (G) Proliferation of the primary and activated OT1-iT and control WT-T cells responding to B16F10-OVA stimulation *in vitro*. The primary and activated OT1-iT cells or control WT-T cells (1×10^5^) labeled with cell proliferation dye eFluor 670 were stimulated with 1×10^5^ B16F10-OVA tumor cells, and the proliferation status of the OT1-iT cells or WT-T cells were analyzed after coculture at day 3, day 5, and day 7. (H) Statistical analysis of CD8^+^eFluor670^+^ primary OT1-iT cells or control WT-T cells in (G), left (n=3). (I) Statistical analysis of CD8^+^eFluor670^+^-activated OT1-iT cells or control WT-T cells in (G), right (n=3). Data are representative of three independent experiments and were analyzed by two-sided independent *t*-test (A, B, D, E, F, H, and I) or Mann-Whitney test (D). *p<0.05, **p<0.01, ***p<0.001. iT, induced T cells; LDH, lactate dehydrogenase; NS, not significant; OD, optical density; OVA, ovalbumin; WT, wild type.

To establish the tumor targets of the OT1-iT cells, we constructed an ovalbumin (OVA)-expressing B16F10 melanoma cell line (B16F10-OVA), which presents the MHC-I restricted OVA antigen. Next, we cocultured SP-derived OT1-iT cells (effector (E) cell) with B16F10-OVA cells (target (T) cell) to examine their antitumor activity. We chose wild-type T cells (WT-T) from C57BL/6 mouse as the negative control for their natural TCR repertoire diversity. Particularly, primary splenic OT1-iT cells (1×10^3^, 1×10^4^, 5×10^4^, 1×10^5^, and 2×10^5^) or splenic WT-T cells (1×10^3^, 1×10^4^, 5×10^4^, 1×10^5^, and 2×10^5^) isolated from OT1-iT-*Rag1^-/-^* or WT mice were cocultured with 1×10^4^ B16F10-OVA cells. The number of B16F10-OVA cells sharply decreased after 36 hours of coculture with the primary OT1-iT cells at various E:T ratios (figure 2A, C upper panel and figure 2D) compared with the WT-T control group (p<0.01, p<0.001). Moreover, CD8^+^ OT1-iT cells exhibited robust proliferation in the presence of the B16F10-OVA cells within 7 days, while control CD8^+^ WT-T cells proliferated much more slowly. This indicated direct tumor cell-stimulated activation of the OT1-iT cells (figure 2G, left panel and figure 2H). To test whether T-cell activation preceding the tumor cell coculture could enhance the tumor-killing ability of the OT1-iT cells, we stimulated the primary OT1-iT cells or the primary WT-T cells with CD3/CD28 antibodies for 4 days *in vitro*. Strikingly, a much lower number of the preactivated OT1-iT cells (1×10^2^, 1×10^3^, 5×10^3^, 1×10^4^, and 2×10^4^) than the primary OT1-iT cells could significantly kill the B16F10-OVA cells (1×10^4^) *in vitro*, whereas the activated WT-T cells still exhibited no-killing behavior even at the highest E:T ratio (2:1) (p<0.01, p<0.001) (figure 2B, C lower panel and figure 2E). The proliferation experiments also showed that the activated OT1-iT cells also proliferated much faster than the activated WT-T cells after coculture with the B16F10-OVA cells (figure 2G, right panel and figure 2I). Moreover, the OT1-iT cells demonstrated significantly efficient cytotoxicity activity over WT-T cells as the E:T ratio increased (figure 2F), and the proliferation of the cocultured B16F10-OVA tumor cells was inhibited in the OT1-iT group (p<0.5) (online supplementary figure S2a and b). Taken together, the OT1-iT cells converted from B cells effectively killed tumor cells *in vitro*.

### OT1-iT cells suppress tumor growth *in vivo*

**Figure 3:**
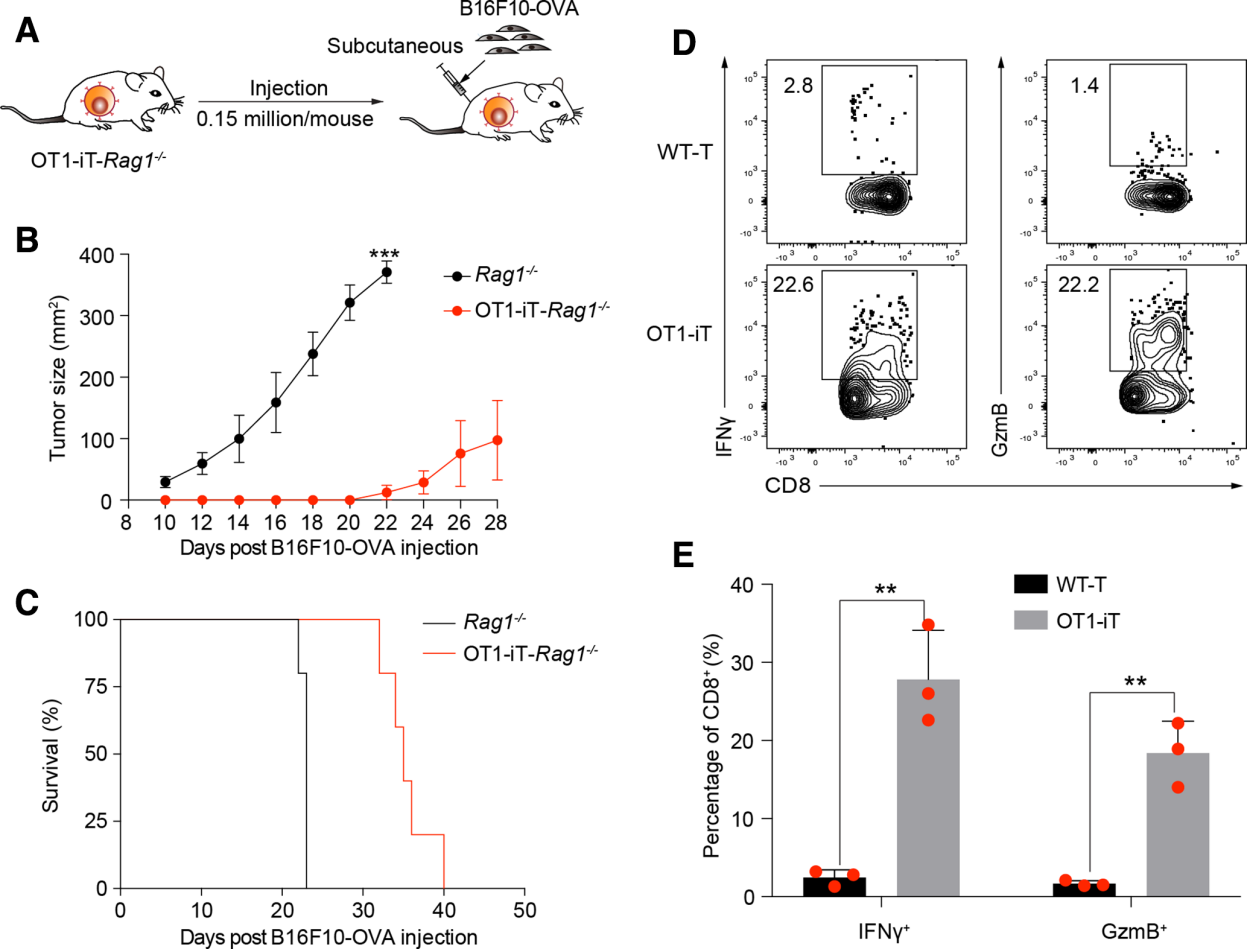
Prevention of the B16F10-OVA tumor cell growth in OT1-iT-*Rag1^-/-^* mice. (A) Schematic diagram of the primary OT1-iT cells for anti-tumor therapy in OT1-iT-*Rag1^-/-^* mice. B16F10-OVA cells (0.15 million/mouse) were subcutaneously injected into the groin of the *Rag1^-/-^* or OT1-iT-*Rag1^-/-^* mice 6 weeks after transplantation with OT1 pro-pre-B cells transduced with *Hoxb5* retroviruses. (B) Tumor growth in *Rag1^-/-^* (control) and OT1-iT- *Rag^-/--^* mice (OT1-iT). The tumor sizes (length×width, mm^2^) were measured using a caliper every other day. Mice with tumor size larger than 400 mm^2^ were euthanized following ethical standard (each group n=5). (C) Kaplan-Meier survival curve of *Rag1^-/-^* (control) or OT1-iT-*Rag1^-/-^* mice post-B16F10-OVA injection (n=5, p=0.0018, log-rank test). (D) Intracellular staining of IFNγ and GzmB in the infiltrated OT1-iT cells. T cells were isolated from the OT1-iT-*Rag1^-/-^* mouse when tumor size reached 400 mm^2^. The IFNγ^+^ or GzmB^+^ population is shown for the CD45.2^+^Mac1^-^Gr1^-^CD8^+^ iT cells. The bone marrow from wild-type (WT) mouse was used as the negative control to define the positive population of IFNγ^+^ or GzmB. Flow plots from one representative mouse of each group are shown. (E) Percentage of IFNγ^+^ or GzmB^+^ CD8^+^ OT1-iT cells and WT-T cells in (D) (n=3). Data are representative of three independent experiments and were analyzed by two-sided-independent *t*-test (B, E). **p<0.01, ***p<0.001. GzmB, granzyme B; IFNγ, interferon gamma; iT, induced T cells; OVA, ovalbumin; WT, wild type.

To evaluate the impact of OT1-iT cells on B16F10-OVA tumor proliferation in OT1-iT-*Rag1^-/-^* mice, B16F10-OVA tumor cells were subcutaneously injected into OT1-iT-*Rag1^-/-^* mice 6 weeks’ post-transplantation (figure 3A). Consequently, the tumor sizes were less than 160 mm^2^ on day 28 post-B16F10-OVA injection, while those in the control (*Rag1^-/-^* transplanted with B16F10-OVA) group were around 400 mm^2^ on day 22 postinjection, leading to euthanasia to comply with experimental animal ethics procedures (figure 3B). Comparatively, the B16F10-OVA tumor-bearing OT1-iT-*Rag1*^-/-^ mice survived up to 40 days (figure 3C), demonstrating a prolonged survival than the untreated control. In addition, we observed that the tumor-infiltrated OT1-iT cells (CD45.2^+^CD8^+^) secreted interferon gamma (IFNγ) and granzyme B (GzmB) (figure 3D, E), which indicated their tumor cell killing behavior. Thus, these results demonstrated that the OT1-iT cells can reduce tumor development in OT1-iT-*Rag1^-/-^* mouse.

**Figure 4:**
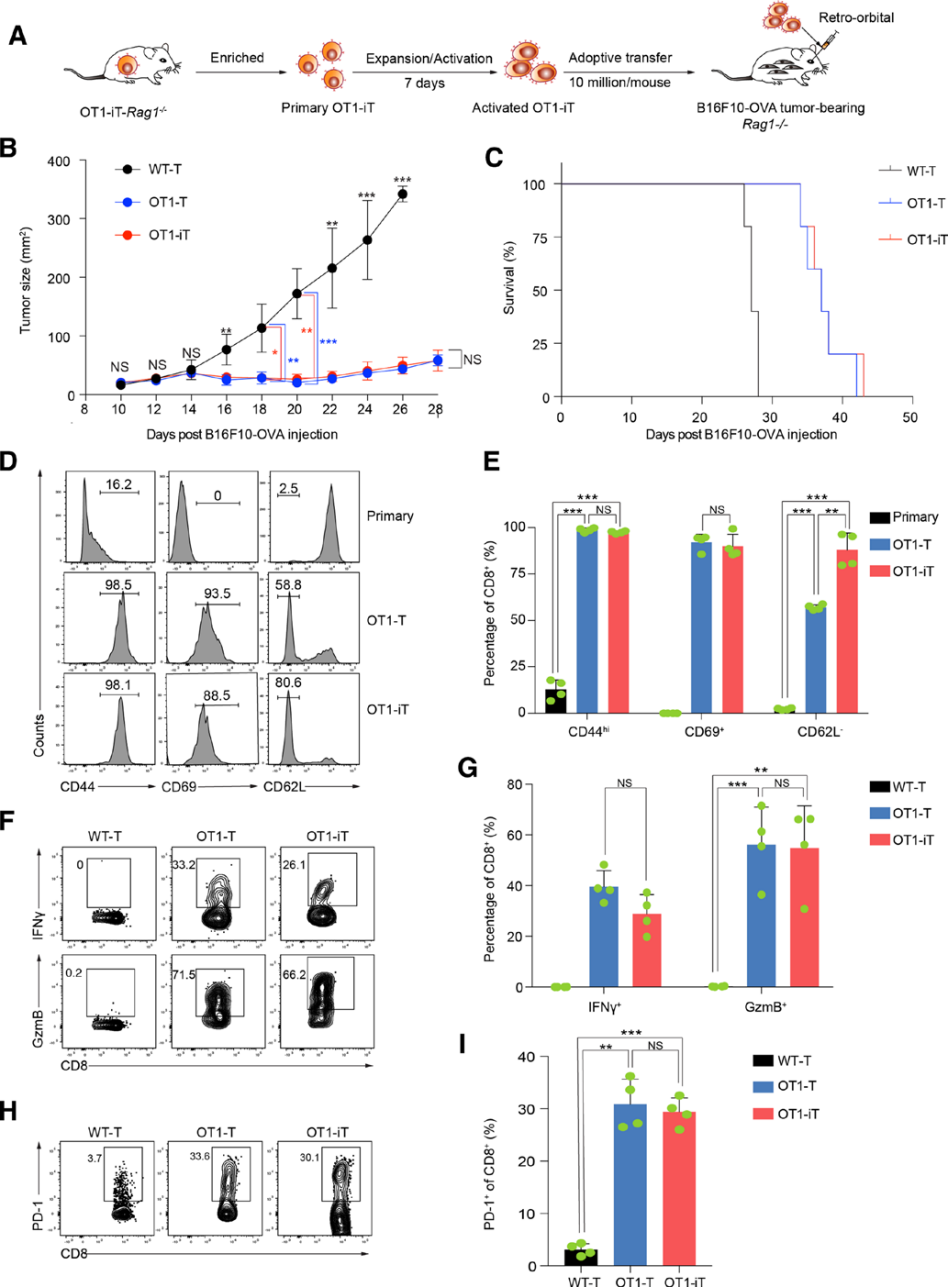
Adoptive transfer of OT1-iT cells relieves the tumor burden *in vivo*. (A) Schematic diagram of the OT1-iT cells for antitumor therapy in the B16F10-OVA tumor-bearing *Rag1^-/-^* mice. *Rag1^-/-^* mice were subcutaneously injected with B16F10-OVA cells (0.15 million/mouse) in the groin to establish the melanoma tumor model. Expanded and activated OT1-iT cells were obtained by stimulating primary OT1-iT cells with anti-CD3/CD28 beads for 7 days. The activated OT1-iT cells (10 million/mouse) were transplanted into the tumor-bearing mice 10 days after the tumor cell injections. (B) Tumor growth in B16F10-OVA tumor-bearing *Rag1^-/-^* mice. The tumor-bearing mice with similar tumor size were randomly divided into three groups and received activated WT-T, OT1-T, or OT1-iT cells (10 million/mouse) 10 days after B16F10-OVA tumor cell injection (n=5 each group). The tumor sizes (length×width, mm^2^) were measured using a caliper every other day. Tumor size data are shown from day 10 to day 28. Mice with tumor sizes larger than 400 mm^2^ were euthanized for ethical consideration. (C) Kaplan-Meier survival curve of the tumor-bearing *Rag1^-/-^* mouse (n=5 each group, p=0.0002, log-rank test). (D) The activation status of the tumor-infiltrating OT1-T and OT1-iT cells. Tumor-bearing *Rag1^-/-^* mice transplanted with the T cells were sacrificed when the tumor size reached 400 mm^2^, and the tumor-infiltrating T cells were isolated from the tumors for further flow cytometric analysis. Flow plots of the CD44^hi^, CD69^+^, and CD62L^-^ population of one representative mouse from each group are shown for the gated CD45.2^+^Mac1^-^Gr1^-^CD8^+^ T cells. Primary T cells isolated from the peripheral blood of OT1-iT-*Rag1^-/-^* mice were used as negative control. (E) Percentage of CD44^hi^, CD69^+^ and CD62L^-^ cells in (D) (n=4). (F) Intracellular staining of IFNγ and GzmB in the tumor-infiltrating OT1-T and OT1-iT cells from treated tumor-bearing *Rag1^-/-^* mice. WT-T cells isolated from the bone marrow of WT mice were used as control. (G) Percentage of IFNγ^+^ and GzmB^+^ populations of WT-T cells, tumor-infiltrating OT1-T, and OT1-iT cells in (F) (n=4). (H) Flow cytometric analysis of PD-1 of WT-T cells, tumor-infiltrating OT1-T, and OT1-iT cells. (I) Percentage of PD-1^+^ cells in (H). Data are representative of three independent experiments and were analyzed by two-sided independent *t*-test (B, E, G, and I) or Mann-Whitney test (E). *p<0.05, **p<0.01, ***p<0.001. GzmB, granzyme B; IFNγ, interferon gamma; iT, induced T cells; NS, not significant; OVA, ovalbumin; WT, wild type.

To mimic the treatment scenario of patients with tumor, we performed an adoptive transfer assay of the SP-derived OT1-T cells (OT1 transgenic mouse, positive control) and OT1-iT cells to allogenic mice bearing tumors. We isolated splenic OT1-T cells from OT1 mice, OT1-iT cells from primary OT1-iT-*Rag1^-/-^* mice, and WT-T cells from the WT mice followed by expansion and activation, and then adoptively transferred them into tumor-bearing mice (figure 4A). As expected, the tumor burden in the OT1-iT cell-treated B16F10-OVA tumor-bearing mice was significantly reduced, and these mice survived up to 43 days post-B16F10-OVA tumor cell injection. This achieved comparable therapeutic effects as the OT1-T cell treatment (up to 42 days). In contrast, tumor sizes of WT-T cell-treated mice reached the limit of ethic allowance within 28 days’ postinjection, resulting in their sacrifice (figure 4B, C). We further analyzed the activation status of the OT1-iT and OT1-T cells infiltrated in the tumors and observed that both the OT1-iT and OT1-T cells (CD45.2^+^CD8^+^) were completely activated (CD44^hi^CD69^+^CD62L^-^), comparing with primary T cells. Notably, OT1-iT cells had more activated CD44^hi^CD69^+^CD62L^-^ cells than OT1-T cells (figure 4D, E). In addition, the activated OT1-iT cells secreted comparable levels of IFNγ and GzmB to OT1-T cells (figure 4F, G), indexing their tumor-eradicating behavior. Furthermore, programmed cell death protein 1 (PD-1) expression was upregulated in the infiltrated OT1-T and OT1-iT cells in the mice with tumor recurrence (figure 4H, I). These results illustrate the antitumor capacity of the reprogrammed OT1-iT cells *in vivo*.

## Discussion

In this study, we generated naive OT1-iT cells in *Rag1^-/-^* mouse from *Hoxb5*-overexpressing pro-pre-B cells. Just as previously reported,^15^ it takes 4 weeks to obtain OT1-iT cells in recipients transplanted with *Hoxb5*-overexpressing pro-pre-B cells, which is much shorter than either HSC-derived or iPSC-derived OT1-iT cells (7–8 weeks).^6 10^ However, the starting pro-pre-B cells in our study were collected from bone marrow, which is an obstacle for further translational research since preparing bone marrow-derived cells were invasive and quantity limited. Thus, new methods need to be developed to obtain abundant pro-pre-B cells *in vitro*, such as via natural hematopoietic stem and progenitor cells (HSPC) differentiation and expansion.^17 18^

Besides the *Hoxb5*-expressing OT1 pro-pre-B cells can generate OT1-iT cells, the WT pro-pre-B cells without expressing OT1 can also transdifferentiate into OT1-iT cells when simultaneously enforcing expression of *Hoxb5* and OT1 TCR (online supplementary figure S3a-c). Moreover, these OT1-iT cells also significantly reduced the tumor burden and prolonged survival (online supplementary figure S3d-e). Of note, this tandem expression approach showed much lower assembling efficiencies of OT1 TCR αβ chains in the CD8^+^ iT cells than by OT1 transgenic method, which is largely due to the optimized construction strategy of the OT1 transgenic mouse.^19^ In addition, endogenous TCRα chains can also have additional rearrangements in the presence of exogenous ones since TCRα loci rearrangements have no allelic exclusion phenomenon.^20 21^ Expectedly, it can improve the efficiency of OT1-iT cells by searching for a stronger promoter or enhancer to competitively express OT1 TCRα chains or directly blocking additional endogenous TCR rearrangements by knockdown of RAG recombinase expression.

We have confirmed that the transdifferentiation-derived OT1-iT cells can prevent tumor growth both in reconstituted OT1-iT-*Rag1^-/-^* and adoptive tumor-bearing models. Alternatively, it is worth trying to directly generate OT1-iT cells in tumor models to evaluate their antitumor ability, as this way mimics natural disease development. In conclusion, we have developed an alternative method of generating tumor-specific iT cells in animals by a *de novo* blood lineage-transdifferentiation approach.

## Materials and methods

### Generation and analysis of the OT1-iT cells

Pro-pre-B cells’ (C57BL/6 mouse) or OT1 pro-pre-B cells’ (OT1 transgenic mouse) isolation, infection, and transfer were performed as previously described.^16^ Briefly, pro-pre-B cells from WT mice or OT1 transgenic mice were first enriched via positive magnetic affinity cell sorter selection using B220-biotin and anti-biotin MicroBeads (Miltenyi Biotec), and then sorted from the enriched B220^+^ cells by Aria III (BD). The sorted cells were subsequently stimulated with the pro-pre-B cell medium for 12–16 hours prior to retroviral transduction. Pro-pre-B cells (*Hoxb5* retrovirus or OT1-*Hoxb5* retrovirus) and OT1 pro-pre-B cells (GFP retrovirus or *Hoxb5* retrovirus) were transduced with retrovirus by two rounds of spin transfection (800 g, 90 min, 35℃) at a density of 1 million/mL. For transplantation, 5 million GFP^+^ pro-pre-B cells or OT1 pro-pre-B cells were injected into the retro-orbital veins of the irradiated *Rag1^-/-^* recipients (3.5 Gy, RS2000; Rad Source). All recipients were given water supplemented with trimethoprim–sulfamethoxazole for 2 weeks to prevent infection. OT1-iT lymphocytes were analyzed 4–6 weeks’ post-transplantation.

### *In vitro* function analysis of the OT1-iT cells

Primary OT1-iT cells or WT-T cells derived from the SP of the OT1-iT-*Rag1^-/-^* mice or C57BL/6 mice were enriched by depletion of Ter119^+^CD11b^+^Gr1^+^B220^+^NK1.1^+^CD11c^+^ cells and cultured in the T-cell medium without interleukin 2 at a density of 1×10^6^/mL. Activated OT-iT cells were obtained by coculturing primary OT1-iT cells with Dynabeads Mouse T-Activator CD3/CD28 (CD3/CD28 Gibco) for T-cell expansion and activation for 4 days. Primary or activated OT1-iT (E) cells were incubated with 1×10^4^ B16F10-OVA (T) cells in 96-well plates for 36 hours at respective E:T ratios (primary T cells, E:T=0.1:1, 1:1, 5:1, 10:1, 20:1; activated T cells, E:T=0.01:1, 0.1:1, 0.5:1, 1:1, 2:1). The number of B16F10-OVA tumor cells (DsRed^+^) were enumerated using the CountBright Absolute Counting Beads (Thermo Fisher) by LSRFortessa-X20 (BD). Microphotographs were taken to assess the antitumor effect of primary or activated OT1-iT using the ImageXpress Micro Confocal (Molecular Devices). The Non-Radioactive Cytotoxicity Assay kit (Promega) was used to analyze the lactate dehydrogenase released from the coculture cells, and the cytotoxicity of the OT1-iT cells were analyzed following the instructions of the assay kit.

### *In vitro* OT-iT cell proliferation assay

The proliferation analysis of the OT1-iT cells was performed as described.^22^ Prior to coculture, OT1-iT cells or WT-T cells were stained with the Cell Proliferation Dye eFluor 670 according to the manufacturer’s directions. The stained T cells and B16F10-OVA were cocultured with T-cell medium. The proliferation status of the OT1-iT cells (CD45.2^+^CD8^+^) were analyzed at day 0, day 3 and day 7 after coculture by the LSRFortessa-X20 (BD).

### B16F10-OVA melanoma tumor model

OT1-iT-*Rag1^-/-^* mice or *Rag1^-/-^* mice, which were used as the tumor model, were transplanted with B16F10-OVA cells (0.15 million/mouse) in the groin by subcutaneous injection. For the OT1-iT-*Rag1^-/-^* tumor model, *Rag1^-/-^* mice were transplanted with the OT1 pro-pre-B cells transduced with *Hoxb5* retroviruses (5 million/mouse) or pro-pre-B cells transduced with OT1-*Hoxb5* retroviruses 6 weeks prior to the B16F10-OVA cell injection. For adoptive transfer, the splenic T cells (WT-T, OT1-T, OT1-iT) were first expanded and activated for 7 days *in vitro* using the Dynabeads Mouse T-Activator CD3/CD28 for T-cell expansion and activation. The expanded and activated T cells (10 million/mouse) were transplanted into the tumor-bearing *Rag1^-/-^* mice 10 days after B16F10-OVA cell injection. The tumor size was measured every other day using calipers and calculated as length ×width (mm^2^). Mice with tumor sizes larger than 400 mm^2^ were euthanized for ethical consideration.

### Infiltrated OT1-iT cell isolation and effector functional analysis

OT1-iT cells were isolated from the melanoma tumors from the tumor-bearing mice as previously described.^23^ The isolated cells were stained with antibodies against CD45.2, CD8a, TCRVα2, and TCRVβ5. For the intracellular staining, cells isolated from the tumors were first stained with the surface antibodies (CD45.2, CD11b, Gr1 and CD8), fixed, and then stained with Allophycocyanin (APC)-conjugated IFNγ and PE-conjugated GzmB.

### Statistical analysis

Flow cytometry data were analyzed by the FlowJo software. Prism7 (GraphPad) and SPSS (V.22.0) were used for the statistical analysis.

Extended experimental procedures including regents, cell culture, retrovirus preparation, and BCR analysis are described in online supplementary file.
