# Supplementary material for: Hematopoietic lineage-converted T cells carrying tumor-associated antigen-recognizing TCRs effectively kill tumor cells
Source: J Immunother Cancer. 2020 Jul 14;8(2):e000498. doi: 10.1136/jitc-2019-000498 (PMC7368548; doi:10.1136/jitc-2019-000498)
Supplement: Supplementary data [file jitc-2019-000498supp001.pdf]

Fig. S1

a

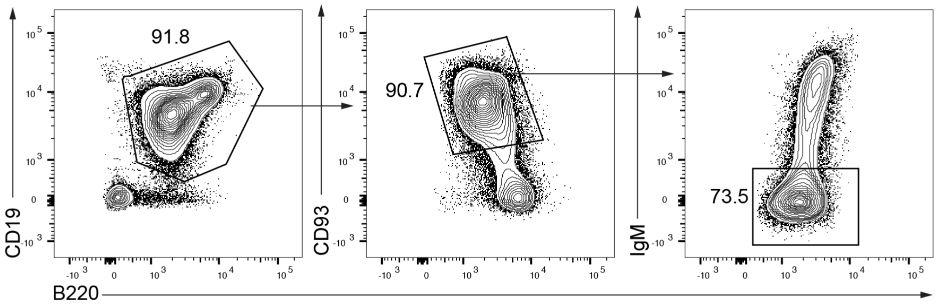

b

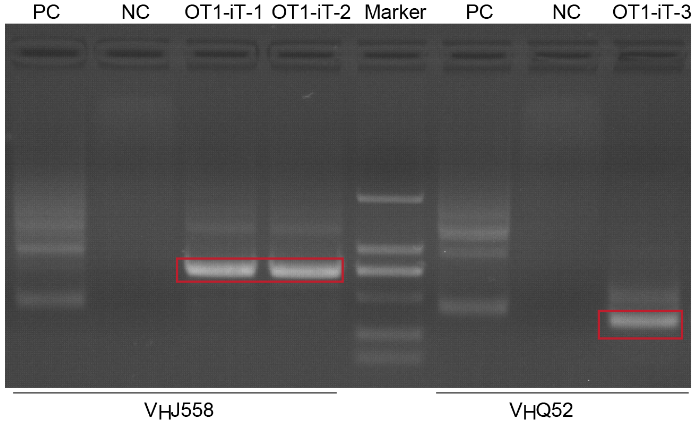

Type	BCR IgH V(D)J rearrangements				
Sample ID	V - name	D - name	J - name	Productive	Ig Blast Identity (%)
OT1-iT-1	IGHV1-64*01	IGHD6-1*01	IGHJ3*01	NO	99.6
OT1-iT-2	IGHV1-61*01	IGHD4-1*01	IGHJ3*01	NO	99.6
OT1-iT-3	IGHV2-6*03	IGHD2-4*01	IGHJ4*01	NO	100

c

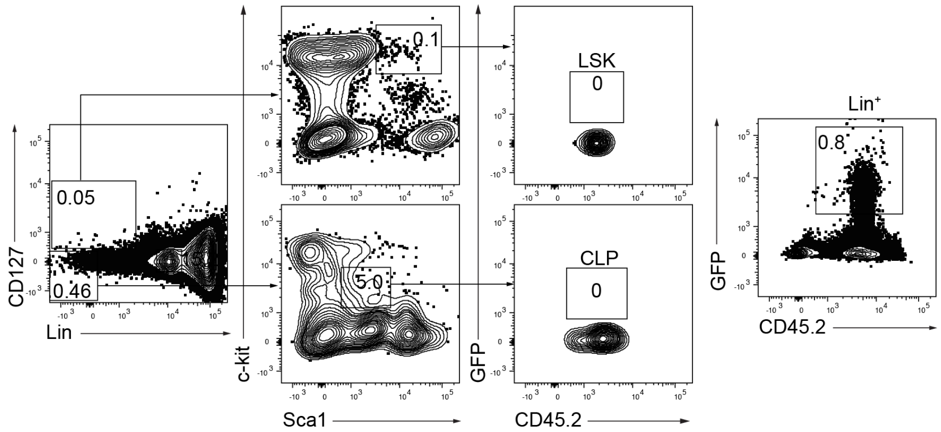

1

Fig. S1 Generation of OT1-iT cells from OT1 pro-pre-B cells, related to Fig. 1. (a) Sorting strategy of the OT1 pro-pre-B cells ($CD3^-Mac1^-Ter119^-B220^+CD19^+CD93^+IgM^-$). (b) PCR bands and Ig blast results of BCR Ig heavy chain V(D)J rearrangements in the single OT1-iT cells. Single OT1-iT cells from the spleen of the OT1-iT-*Rag1*$^{-/-}$mouse six weeks post-transplantation were sorted and the genome were extracted and amplified for BCR analysis. (c) Flow cytometry analysis of the donor derived common lymphoid progenitor (CLP) and $Lin^+Sca1^+c\text{-}kit^+$ (LSK) cells in the bone marrow (BM) of the *Rag1*$^{-/-}$ recipients 6 weeks after transplantation with OT1 pro-pre-B cells transduced with *Hoxb5* retroviruses. Donor derived CLP cells were defined as $Lin^-CD127^+Sca1^{mid}c\text{-}kit^{mid}CD45.2^+GFP^+$, and LSK cells were defined as $Lin^-Sca1^+c\text{-}kit^+CD45.2^+GFP^+$.

Hu F, *et al. J Immunother Cancer* 2020; 8:e000498. doi: 10.1136/jitc-2019-000498

Fig. S2

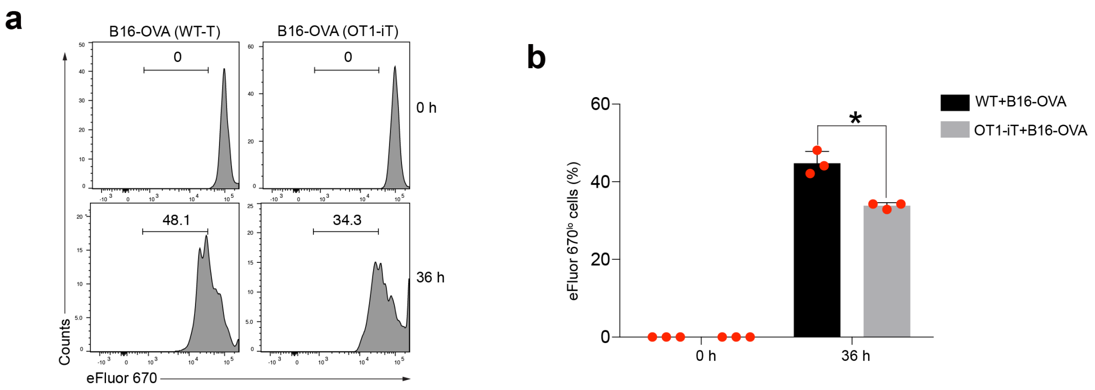

Fig. S2. Proliferation assay of B16F10-OVA cells, related to Fig.2. (a) Tumor cells stained with eFluor670 were analyzed prior to and after 36 hours co-culture with WT-T or OT1-iT cells. (b) Percentage of eFluor670^{lo} cells in (a) (n = 3). Data are representative of three independent experiments and were analyzed by two-sided-independent *t-test.* *, $p < 0.05$.

Fig. S3

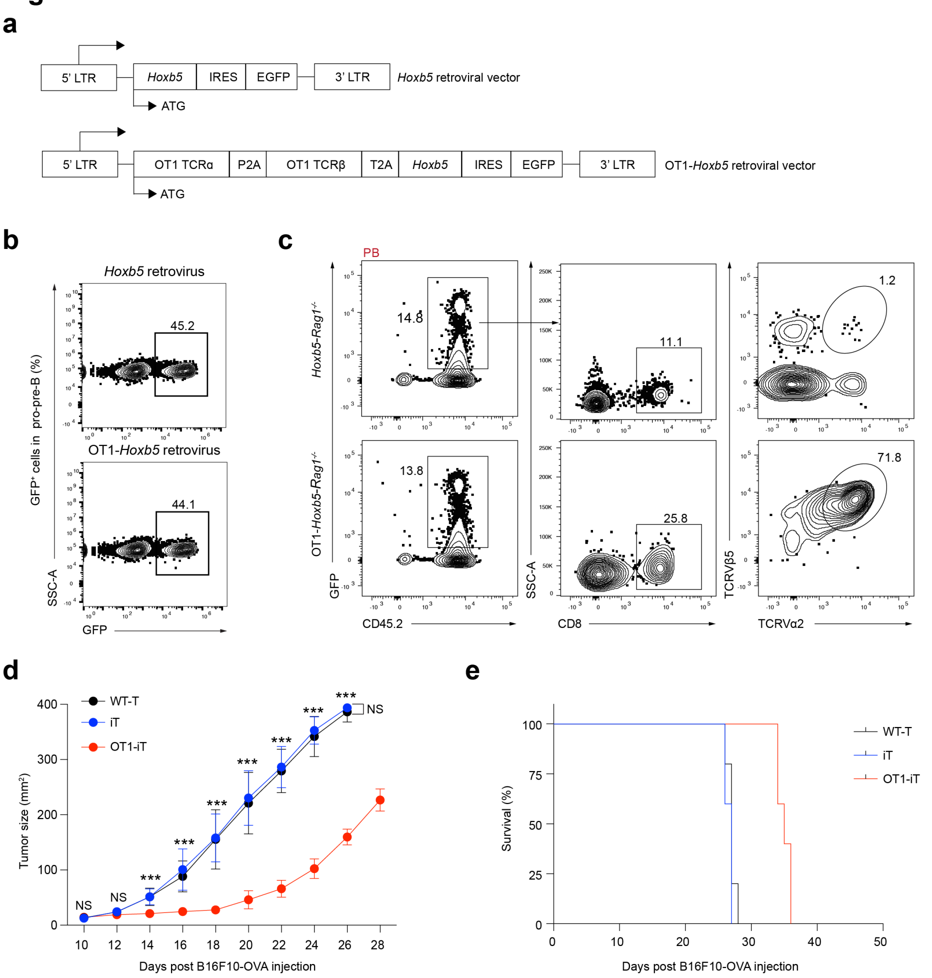

Fig. S3. OT1-iT cells derived from pro-pre-B transduced with OT1-*Hoxb5* retrovirus exhibit the anti-tumor capacity. (a) Strategy of the construction of OT1-*Hoxb5* retrovirus vector. The element encoding OT1 and *Hoxb5* was cloned into the pMYs-IRES-GFP retroviral vector. (b) The infection ratio of the pro-pre-B cells post OT1-*Hoxb5* retrovirus or *Hoxb5* retrovirus transduction. (c) OT1-TCR analysis of the PB derived of GFP^{+}CD8^{+} cells in *Rag1*$^{-/-}$ mice four weeks after transplanting with *Hoxb5*-retrovirus pro-pre-B cells (*Hoxb5-Rag1*$^{-/-}$) or OT1-*Hoxb5*-retrovirus pro-pre-B cells (*OT1-Hoxb5-Rag1*$^{-/-}$). (d) Tumor growth in *Rag1*$^{-/-}$ mouse. *Rag1*$^{-/-}$ mice were randomly divided into three groups 10 days after B16F10-OVA injection. The expanded WT-T, iT (*Hoxb5-Rag1*$^{-/-}$) and OT1-iT cells (*OT1-Hoxb5-Rag1*$^{-/-}$) (10

Hu F, *et al. J Immunother Cancer* 2020; 8:e000498. doi: 10.1136/jitc-2019-000498

million/mouse) were separately transferred into the tumor-bearing mouse of each group (n = 5). Tumor sizes (length × width) were measured every other day. Two-sided-independent test, NS, not significant, ***, $p < 0.001$. (e) Kaplan-Meier survival curve of the tumor-bearing *Rag1*$^{-/-}$ mouse (n = 5 each group, $p = 0.0014$, long-rank test).

Supplementary materials and methods

Mice and reagents

C57BL/6J(B6) mice (referred as WT) were purchased from Bejing Vital River Laboratory Animal Technology. *Rag1*$^{-/-}$ mice (C57BL/6 background) were a gift from Dr. Z. Liu from Institute of Biophysics (CAS, China). OT1 transgenic mice were kindly provided by Dr. Penghui Zhou (SYSUCC, China). All mice were housed in the SPF-grade animal facility of the Guangzhou Institutes of Biomedicine and Health, Chinese Academy of Sciences (GIBH, CAS, China). All animal experiments were approved by the Institutional Animal Care and Use Committee of Guangzhou Institutes of Biomedicine and Health (IACUC-GIBH). eBioscience™ Cell Proliferation Dye eFluor™ 670 and antibodies of CD45.2 (104), NK1.1 (PK136), CD11c (N418), B220 (6B2), CD19 (6D5), CD93 (PB.493), IgM (II/41), CD2 (RM2-5), CD3 (145-2C11), CD4 (RM4-5), CD8 (53-6.7), Gr1 (RB6-8C5), Mac1 (M1/70), Ter119 (TER-119), TCRVα2 (B20.1), TCRVβ5 (MR9-4), CD25 (PC61), CD44 (IM7), Sca-1 (E13-161.7), c-kit (2B8), CD127 (A7R34), TNFα (MP6-XT22), IFNγ (DB-1), and GzmB (OA16A02) were brought from eBioscience or biolegend.

Cell culture

HEK293T (ATCC) and Plat-E (Cell Biolabs, Inc) cells were maintained in DMEM/high glucose (Hyclone) supplemented with 10% FBS (Natocor). B16F10 melanoma cell line was purchased from the Cell Bank of Chinese Academy of Sciences. B16F10-OVA cells were established with a lentivirus expressing the chicken OVA cDNA and DsRed reporter. B16F10

Hu F, *et al. J Immunother Cancer* 2020; 8:e000498. doi: 10.1136/jitc-2019-000498

and B16F10-OVA cell line were cultured in RPMI 1640 (Gibco) supplemented with 10% FBS (Natocor), 1% GlutaMAX (Gibco) and 1% sodium pyruvate (Gibco). Pro-pre-B cells were cultured with the medium of opti-MEM (Gibco) supplemented with 15% FBS (Gibco), 1% GlutaMAX (Gibco), 10^{-4} M β-ME (Sigma), 10 ng/ml SCF (Peprotech), 10 ng/ml Flt3L (Peprotech) and 10 ng/ml IL7 (Peprotech). T cells were cultured with the medium of RPMI-1640 (Gibco) supplemented with 10% FBS (Gibco), 1% GlutaMAX (Gibco), 1% NEAA (Gibco), 10^{-4} M β-ME (Sigma), 100 IU/ml recombinant human IL-2 (Peprotech) and 2 ng/ml murine IL-7 (Peprotech).

Construction and generation of OT1-*Hoxb5* retrovirus.

The cDNA of murine OT1 TCR (TCRα-P2A-TCRβ) (OT1-2A.pMIG II, Addgene) and *Hoxb5* were inserted into pMYs-IRES-EGFP (RTV-021, Cell Biolabs, INC) to generate a pMYs-TCRα-P2A-TCRβ-T2A-*Hoxb5*-IRES-EGFP recombinant vector. Retroviruses was generated using Plat-E cells (containing retro-virus packaging elements) by calcium phosphate method.

Analysis of immunoglobulin heavy-chain V(D)J rearrangement

The sorted single splenic OT1-iT cells ($CD45.2^+GFP^+CD8^+TCRV\alpha2^+TCRV\beta5^+$) from the OT1-iT-*Rag1*$^{-/-}$ mouse were used for genome extraction, and the amplified genome was used as the template for IgH V(D)J rearrangement analysis.

Hu F, *et al. J Immunother Cancer* 2020; 8:e000498. doi: 10.1136/jitc-2019-000498
